# Successful use of trametinib and dasatinib combined with chemotherapy in the treatment of Ph-positive B-cell acute lymphoblastic leukemia: A case report

**DOI:** 10.1097/MD.0000000000026440

**Published:** 2021-06-25

**Authors:** Jing Wang, Shu-Hong Shen, Bin-Fei Hu, Guan-Ling Wang

**Affiliations:** a Ningbo Women and Children's Hospital, Ningbo; b Shanghai Children's Medical Center, Shanghai Jiaotong University, Shanghai, China.

**Keywords:** acute lymphoblastic leukemia, case report, chemotherapy, targeted drug, trametinib

## Abstract

**Rationale:**

Relapsed or refractory acute lymphoblastic leukemia poses a significant clinical challenge due to its poor prognosis, showing survival rates of less than a year even with the use of novel therapies. In this report, we describe the safe and effective use of trametinib combined with dasatinib in a patient with acute lymphoblastic leukemia (ALL). To the best of our knowledge, this is the first report on the successful use of 2 targeted drugs such as trametinib and dasatinib in a pediatric patient with Ph+ ALL and recurrent pancreatitis.

**Patient concerns:**

A 6-year-old boy with ALL and Philadelphia chromosome (Ph+) who had recurrent asparaginase-associated pancreatitis.

**Diagnosis:**

The patient was diagnosed with ALL, based on clinical features, laboratory analyses, bone marrow aspiration evaluation in morphology, immunology, cytogenetics, and molecular.

**Interventions:**

The patient was treated with dasatinib combined with an intermediate risk-oriented chemotherapy. However, owing to recurrent asparaginase-associated pancreatitis, the patient has to abandon asparaginase in consolidation. Considering the high risk of relapse, we used trametinib and dasatinib combined with chemotherapy as maintenance chemotherapy.

**Outcomes:**

After 6 months, there were no obvious side effects or residual disease.

**Lessons:**

We suggest that the combination of trametinib and dasatinib may represent a viable option to treat patients with potential relapsed/refractory Ph+ ALL.

## 1 Introduction

Introduction of tyrosine kinase inhibitors (TKIs) has significantly improved the management and prognosis of patients with the Philadelphia chromosome (Ph+) and acute lymphoblastic leukemia (ALL). Complete response rates range between 91% and 98%, with an unprecedented 4-year overall survival rate of up to 71%.^[1]^ The relapse rate of pediatric patients with Ph+ ALL is high when they receive an inadequate course of the planned asparaginase therapy. The outcomes of these patients are inferior compared with those of patients that receive adequate doses of the planned therapy.^[2]^

Some data indicate that inhibition of the mitogen-activated protein kinase pathway increases chemosensitivity to glucocorticoids and possibly to other agents, making it an attractive target for the prevention and/or treatment of relapsed disease.^[3]^ Here, we report a very rare case of Ph+ ALL and recurrent pancreatitis treated with trametinib combined with dasatinib. Furthermore, we demonstrate the safe and effective use of these targeted drugs in a pediatric patient.

## 2 Case report

A 6-year-old boy was admitted to our hospital with fever and shoulder pain in September 2018. Physical examination revealed splenomegaly. Laboratory analyses showed the following results: hemoglobin 157 g/L, platelet count 132 × 10^9^/L, white blood cell count (WBC) 75.8 × 10^9^/L. A peripheral blood smear evaluation revealed a large number of immature cells. Bone marrow aspiration evaluation demonstrated an immature B-cell immunophenotype in 92% of the lymphoblasts. Cytogenetic studies revealed the following karyotype: 45XY,-3,-7,der (9) add(9)(p24)t(9;22)(q34;q11.2), der(22)t(9;22),+mar (8)/46, XY(12). Reverse transcription polymerase chain reaction analysis showed a positive result for the BCR-ABL *P190* mutation.

The patient was treated according to the protocol of the Chinese Children's Cancer Group study ALL-2015 with the following drugs as induction therapy: vincristine, 2.4 mg iv on days 1, 8, 15, and 21; idarubicin, 6.4 mg ivgtt on days 5 and 12; prednisone, 35 mg orally daily; and perasparaginase 1600 IU im on days 12 and 26. In addition, TKI dasatinib 65 mg was administered orally daily from the time we were informed that the Philadelphia chromosome was positive.

Post-induction bone marrow aspiration evaluation demonstrated complete remission when assessing minimal residual disease (MRD) on days 19 and 46. Subsequently, the patient received a CAT (cyclophosphamide, cytarabine, and azathioprine) regimen consisting of cyclophosphamide, 0.8 g on day 1; cytarabine 80 mg on days 1 to 7; and azathiopurine, 40 mg on days 1 to 7, as first consolidation treatment. Unexpectedly, the patient developed severe pancreatitis after chemotherapy. Therefore, we switched to asparaginase Erwinia at a dose of 10,000 IU/m^2^ twice weekly after the pancreatitis was under control. During the third asparaginase Erwinia dose, a mild pancreatitis recurred. To avoid a third pancreatitis episode, both perasparaginase and asparaginase Erwinia were excluded from the protocol.

On April 2020, MRD assessment showed 0.01% positive cells. The patient's parents refused bone marrow transplantation because the transplant risk was relatively high. To prevent a relapse, we obtained informed consent from the patient's parents to add trametinib to the maintenance chemotherapy regimen. Details were as follows: trametinib, 0.025 mg/kg on days 1 to 14 every 4 weeks for 18 cycles orally; maintenance therapy: dexamethasone 6 mg/m^2^ on days 1 to 7 every 8 weeks; cyclophosphamidum, 300 mg/m^2^ on day 1; cytarabine, 300 mg/m^2^ on day 1; and amethopterin, 25 mg/m^2^ weekly. During this period, echocardiographic examination was performed every 3 months. Routine and biochemical blood tests were performed regularly every 1 to 3 weeks.

**Figure 1:**
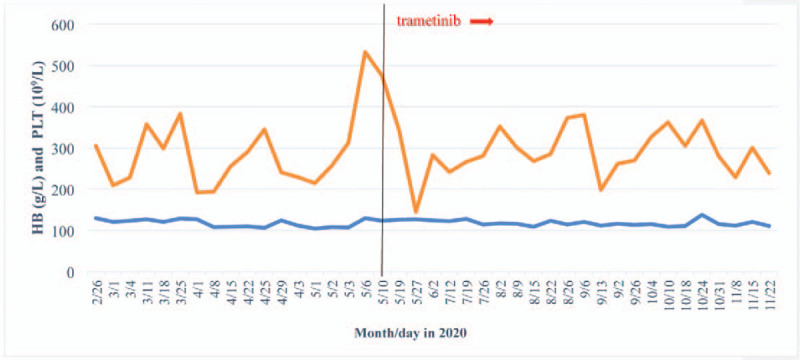
Changes in hemoglobin (HB) level and platelet (PLT) count before and after the introduction of trametinib. Blue curve: changes in HB levels. Orange curve: changes in PLT count. The black vertical line separates the period before and after trametinib introduction.

**Figure 2:**
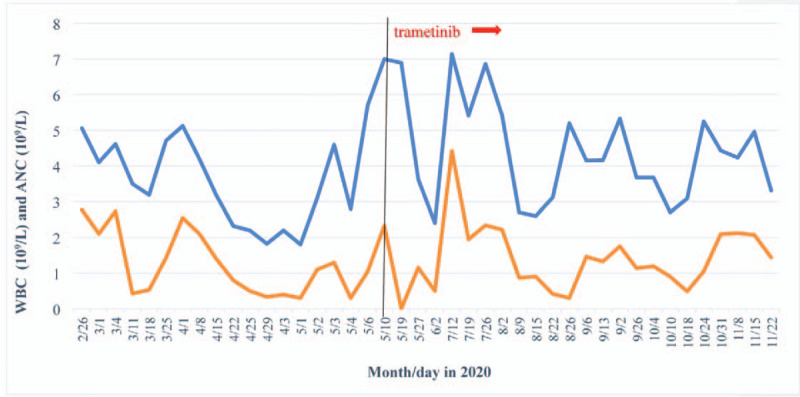
Changes in white blood cell (WBC) count and absolute neutrophil count (ANC) before and after the introduction of trametinib. Blue curve: changes in WBC count. Orange curve: changes in ANC. The black vertical line separates the period before and after trametinib introduction.

**Figure 3:**
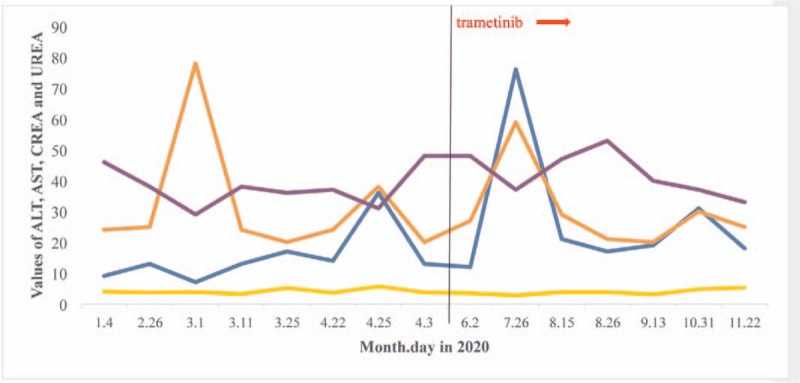
Changes in alanine aminotransferase (ALT), aspartate aminotransferase (AST), creatinine (CREA), and urea levels before and after the introduction of trametinib. Blue curve: changes in ALT levels. Orange curve: changes in AST levels. Purple curve: changes in CREA levels. Yellow curve: changes in urea levels. The black vertical line separates the period before and after trametinib introduction.

**Figure 4:**
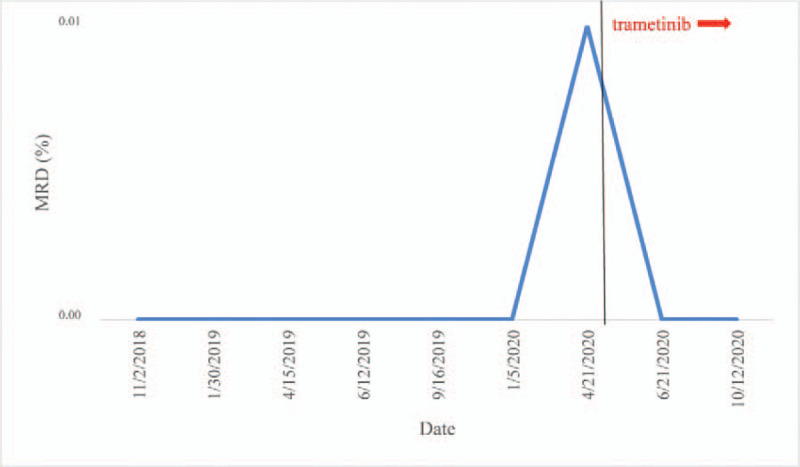
Changes in minimal residual disease (MRD) levels from the first to the most recent morphological complete remission. The black vertical line marks the date trametinib was introduced.

Surprisingly, there was no significant hematologic toxicity after 12 months of treatment. The MRD has always been negative. He has been going to school and lived a normal life. During this time, there were only 2 mild infective fever episodes. Now, let me report his laboratory examinations. In peripheral blood, hemoglobin was above 100 g/L and platelet count was higher than 100 × 10^9^/L when measured at all timepoints (Fig. 1). After using trametinib, the WBC fluctuated between 2.4 and 7.14 × 10^9^/L. Moreover, WBC remained above 3 × 10^9^/L more frequently after using trametinib than before introducing the drug (83%=20/24 vs 77% = 14/18). The absolute neutrophil count (ANC) fluctuated between 0 and 4.42 × 10^9^/L, only falling below 0.5 × 10^9^/L in 4 instances. Moreover, ANC remained above 0.5 × 10^9^/L more frequently after using trametinib than before introducing the drug (83% = 20/24 vs 72% = 13/18) (Fig. 2). Glutamic amino transferase and aspartic amino transferase serum values were 15.25 and 27.714 U/L before using trametinib, and 31.625 and 27 U/L after using trametinib, respectively. Creatinine and urea concentrations were 37.87 vs 42 μmol/L and 4.125 vs 3.9 mmol/L before and after using trametinib, respectively (Fig. 3). During the trametinib regime, 2 echocardiographic evaluations were performed. Cardiac ejection fraction was 60% and 66%. MRD assessment was performed every 3 months, showing negative results (Fig. 4).

## 3 Discussion

According to the extent of asparaginase exposure, previous studies reported that asparaginase-associated pancreatitis (AAP) may occur in 11% of pediatric patients with ALL.^[4–7]^ Although premature asparaginase discontinuation may decrease cure rates,^[7,8]^ AAP represents one of the most common reasons for asparaginase withdrawal in children. Moreover, many pediatric patients experience AAP recurrence after asparaginase reintroduction.^[4,7,9]^ Herein, we described a case of Ph+ ALL in which the patients developed AAP twice. The patient was treated with dasatinib combined with an intermediate risk-oriented induction therapy, achieving a molecular complete remission on day 19. The patient received 2 doses of perasparaginase and 3 doses of asparaginase Erwinia. Using this approach, MRD assessment always resulted negative. Fifteen months later, the MRD assessment result was 0.01%. Considering that the patient was Ph+ and refused bone marrow transplantation, increasing his risk of relapse, we decided to add trametinib to the protocol.

Although the introduction of TKIs has revolutionized the outcomes of Ph+ ALL patients, a proportion of these patients are still unable to achieve a long-term cure because of drug resistance, even when performing hematologic stem cell transplantation. However, with the advent of other novel agents, we are entering an era where we hope to reduce the reliance on transplantation in this disease.^[10]^

In the present case report, we demonstrated that the mitogen-activated protein kinase (MEK) inhibitor trametinib combined with dasatinib display a safe and effective treatment profile in patients with Ph+ ALL. First, MEK inhibition may overcome TKI resistance. The substitution of threonine for isoleucine at residue 315 in Abl (BCR-ABLT315I) forms a protein that is tolerant to almost all TKIs because Bcr-Abl inhibitors do not stimulate the BCR-ABLT315I pathway, whereas TKIs and MEK inhibitors act synergistically to inhibit the growth of BCR-ABLT315I Ba/F3 cells. This induces a synthetic lethality in drug-resistant chronic myeloid leukemia in mice^[11]^ and represents a beneficial therapeutic strategy in patients with AML.^[12]^ Trametinib in combination with TKIs may prevent the development of drug resistance through its regulatory molecule MEK. As suggested in other studies,^[13,14]^ the addition of submaximal concentrations of a MEK inhibitor halts ERK reactivation and sensitizes cells to TKI treatment, resulting in a synergistic combination.^[13]^

ALL patients with poor prognosis, such as infant ALL and Ph+ ALL, tend to have poor clinical response to glucocorticoid agonists.^[15,16]^ Recently, some studies found that trametinib displayed powerful anti-leukemic effects against ALL cells with RAS mutation and MLL rearrangement, as MEK inhibition enhances prednisolone sensitivity.^[17]^ In addition, Jones et al^[3]^ supported the use of trametinib as a potential treatment for relapsed ALL. They suggested that the inhibition of the MEK1/2 pathway combined with chemotherapy not only enhances cell death in relapsed ALL but also overcomes human bone marrow stromal cell protection, which is imperative for therapeutic success in vivo. Moreover, the combination of MEK1/2 inhibitors with traditional cytotoxic chemotherapy may counteract resistance to chemotherapy in relapsed ALL, as MEK2 knockdown or inhibition increases sensitivity to chemotherapy in a p53-dependent manner.^[3]^ Although these experiments were performed in vitro, ex vivo, and in vivo in mice, pediatric clinical trials are underway and could expedite the clinical application of these MEK inhibitors in Ph+ ALL.

In our case, we demonstrated that the drug combination did not display any significant enhancement of cytotoxic activity compared with dasatinib alone.^[18]^ After including trametinib in the treatment regimen, WBC and ANC remained above 3 × 10^9^/L and 0.5 × 10^9^/L, respectively, more frequently than before introducing the drug (77% vs 83% and 72% vs 83%, respectively); this may be related to an infection fever just before trametinib treatment. The mean values of glutamic amino transferase, aspartic amino transferase, and creatinine were slightly higher after the combined treatment with trametinib, while those of urea were similar. Thus, the combination therapy had an increased toxic effect on hepatic and renal function. However, the clinical significance of these differences remains uncertain, requiring studies with a long-term follow-up.

In conclusion, our findings support the use of trametinib as a potential treatment for relapsed ALL. Other studies, such as large sample multicenter studies, are needed to further elucidate the effectiveness and safety of this treatment regimen.

## Acknowledgments

We would like to express our gratitude to Professor Shu-hong Shen of Shanghai Children's Medical Center, for his valuable advice.

## Author contributions

GLW analyzed and interpreted the patient data regarding the hematological disease. JW performed data collection, and was a major contributor in writing the manuscript. BFH and SHS confirm the authenticity of all raw data. All authors read and approved the final manuscript.

**Data curation:** Bin-Fei Hu.

**Investigation:** Shu-Hong Shen, Guan-Ling Wang.

**Resources:** jing wang, Bin-Fei Hu, Guan-Ling Wang.

**Supervision:** Shu-Hong Shen, Bin-Fei Hu.

**Writing – original draft:** jing wang.

**Writing – review & editing:** Shu-Hong Shen, Bin-Fei Hu.
